# Impact of trastuzumab deruxtecan (T-DXd) and brain stereotactic radiosurgery on intracranial control and radionecrosis risk in HER2-positive or -low breast cancer brain metastases

**DOI:** 10.1016/j.breast.2026.104751

**Published:** 2026-03-17

**Authors:** Seok-Joo Chun, Kyubo Kim, Won Ick Chang, Yong Bae Kim, Sun Ha Paek, Kyung-Hun Lee, Jin-Ho Song, Ji Hyun Hong, Jieun Lee, Won Il Jang, Tae Hyun Kim, Kyung Hwan Shin

**Affiliations:** a Department of Radiation Oncology, Dongguk University Ilsan Hospital, Dongguk University College of Medicine, Goyang, South Korea; b Department of Radiation Oncology, Seoul National University Bundang Hospital, Seoul National University College of Medicine, Seongnam, South Korea; c Department of Radiation Oncology, Seoul National University Hospital, Seoul National University College of Medicine, Seoul, South Korea; d Department of Radiation Oncology, Yonsei Cancer Center, Yonsei University College of Medicine, Seoul, South Korea; e Department of Neurosurgery, Seoul National University Hospital, Seoul National University College of Medicine, Seoul, South Korea; f Department of Internal Medicine, Seoul National University Hospital, Seoul National University College of Medicine, Seoul, South Korea; g Department of Radiation Oncology, Seoul St. Mary's Hospital, The Catholic University of Korea College of Medicine, Seoul, South Korea; h Division of Medical Oncology, Department of Internal Medicine, Seoul St. Mary's Hospital, The Catholic University of Korea College of Medicine, Seoul, South Korea; i Department of Radiation Oncology, Korea Institute of Radiological & Medical Sciences, Seoul, South Korea; j Center for Proton Therapy, National Cancer Center, Goyang, South Korea

**Keywords:** Trastuzumab deruxtecan (T-DXd), Stereotactic radiosurgery (SRS), HER2, Breast cancer, Radionecrosis

## Abstract

**Background:**

While trastuzumab deruxtecan (T-DXd) demonstrates intracranial efficacy, the potential for radionecrosis (RN) when combined with stereotactic radiosurgery (SRS) remains a concern, given the established risk with other antibody-drug conjugates like T-DM1. This study evaluated the safety and efficacy of T-DXd and SRS in patients with HER2-positive or -low breast cancer brain metastases (BCBM).

**Methods:**

We conducted a multi-center retrospective analysis of 113 patients (461 SRS treatments) treated with SRS and anti-HER2 agents. Patients were stratified into T-DXd(+) (n = 29 patients, 61 treatments) and T-DXd(−) (n = 84 patients, 400 treatments) groups. Endpoints included RN, radionecrosis-free survival (RNFS), and intracranial control outcomes (any intracranial progression, local failure, and distant intracranial metastasis).

**Results:**

No cases of RN were observed in the T-DXd(+) group, compared with 11 cases in the T-DXd(−) group (p = 0.028). On multivariate analysis, T-DXd(+) status remained significantly associated with improved RNFS (HR 0.31, *p* = 0.009). In the treatment-level analysis, the 1-year cumulative incidence of RN was 0% for T-DXd(+) versus 4.3% for T-DXd(−) (*p* = 0.009). Additionally, T-DXd(+) was associated with significantly better 1-year outcomes for any intracranial progression (40% vs. 76%, *p* < 0.001), local failure (6.6% vs. 29%, *p* = 0.002), and distant intracranial metastasis (40% vs. 66%, *p* = 0.009). All efficacy endpoints remained significant on multivariate analysis.

**Conclusion:**

Combining T-DXd with SRS demonstrated a favorable safety profile without increasing the risk of radionecrosis. Furthermore, this combination was associated with superior intracranial control, encompassing both local and distant outcomes, supporting the potential of T-DXd combined with SRS as an effective and well-tolerated approach for HER2-positive or -low BCBM.

## Highlights

• T-DXd combined with SRS is not associated with an increased risk of radionecrosis.
• T-DXd and SRS is associated with improved intracranial control outcomes.
• T-DXd is associated with decreased local and distant brain failure.
• T-DXd plus SRS could be a safe and effective strategy for HER2+ or low BCBM.

## 1 Introduction

Human epidermal growth factor receptor 2 (HER2)-positive breast cancer is historically associated with aggressive behavior and a high propensity for central nervous system (CNS) dissemination, with about 30% of patients eventually developing brain metastases in the metastatic setting [1]. The management of HER2-positive breast cancer brain metastases (BCBM) has recently undergone a paradigm shift with the advent of next-generation antibody-drug conjugates (ADCs). Trastuzumab deruxtecan (T-DXd), an ADC composed of a HER2-directed antibody linked to a topoisomerase I inhibitor payload, has demonstrated remarkable intracranial efficacy and systemic disease control [2,3], establishing itself as a standard second-line treatment option [4,5].

Despite the superior intracranial activity of T-DXd, stereotactic radiosurgery (SRS) remains a cornerstone of local control for BCBM. Consequently, an increasing number of patients are receiving T-DXd and SRS either concurrently or sequentially. However, the combination of ADCs and high-dose focal radiation has raised significant safety concerns regarding radionecrosis (RN) [[6], [7], [8]]. This concern is largely extrapolated from experiences with trastuzumab emtansine (T-DM1), another HER2-directed ADC carrying a microtubule inhibitor payload. Multiple studies have consistently identified T-DM1 as a significant risk factor for RN when combined with SRS, with reported incidence rates notably higher than those of SRS alone or SRS with other systemic agents [[9], [10], [11], [12]].

The established toxicity profile of T-DM1 has prompted safety investigations to expand beyond T-DM1 to other ADCs in the setting of focal brain irradiation [[6], [7], [8]]. Within this context, T-DXd has emerged as a focal point of interest, given its rapidly expanding role in the management of HER2-positive and HER2-low BCBM. Unlike T-DM1, T-DXd is distinguished by its unique payload mechanism and higher drug-to-antibody ratio [13]. These structural attributes suggest a dual potential: enhanced intracranial tumor control through therapeutic synergy, while simultaneously conferring a distinct risk profile for RN. Although prior experience with T-DM1 has raised concerns that radiosensitization may be associated with increased RN, the clinical implications of these effects in the context of T-DXd remain incompletely understood. Consequently, clinicians are faced with the challenge of balancing intracranial efficacy against potential safety risks. Nevertheless, real-world data specifically evaluating the combination of T-DXd and SRS remain limited [14], and the safety–efficacy trade-off of this approach has yet to be systematically characterized in large, multicenter cohorts.

Given the rapid adoption of T-DXd in clinical practice and the potential morbidity associated with RN, there is an urgent need to clarify the safety and efficacy of this combination. Therefore, we conducted a multi-center retrospective analysis to evaluate the incidence of RN and intracranial efficacy in patients with HER2-positive or HER2-low BCBM treated with T-DXd and SRS, comparing outcomes with those of patients treated with other anti-HER2 regimens.

## 2 Materials and methods

### 2.1 Study design and patient selection

This multi-center retrospective study was conducted across five medical centers in South Korea. We identified patients with HER2-positive or HER2-low breast cancer who underwent SRS for brain metastases between January 2019 and December 2024.

The inclusion criteria were as follows: (1) histologically confirmed HER2-positive or HER2-low breast cancer; (2) diagnosis of brain metastases treated with SRS; and (3) administration of HER2-directed systemic therapies, such as T-DXd, T-DM1, trastuzumab, pertuzumab, or lapatinib, during the clinical course. HER2-low status was defined according to the ASCO/CAP guidelines (immunohistochemistry [IHC] 1+ or IHC 2+ with negative in situ hybridization).

Exclusion criteria included: (1) unavailability of detailed dosimetric data for SRS (e.g., number of target lesions, prescribed dose, treatment volume); or (2) absence of follow-up brain magnetic resonance imaging (MRI) after SRS, which precluded the assessment of treatment response or RN.

### 2.2 Treatment and patient stratification

In accordance with the inclusion criteria, all patients received both SRS and HER2-directed systemic therapy. For each SRS course, detailed dosimetric parameters were collected, including treatment volume, prescription dose, and fractionation scheme. SRS was classified as either single-fraction or fractionated SRS. Single-fraction SRS consisted of a single treatment session, whereas fractionated SRS was defined as stereotactic radiotherapy delivered in multiple fractions. *Multiple SRS courses* were defined as SRS delivered in separate treatment episodes, distinct from fractionated SRS. Additionally, repeated irradiation delivered to a previously treated lesion (in-field recurrence) was defined as *repeated SRS*. A comprehensive history of systemic treatments was also recorded, with specific attention to the administration of T-DM1 and T-DXd.

To accurately evaluate the impact of T-DXd, patients were stratified based on the timing of drug administration relative to the specific endpoint being analyzed. A treatment was classified as the T-DXd(+) group if T-DXd was initiated before the occurrence of the event of interest (RN or intracranial progression) or before the last follow-up. Conversely, if an event occurred prior to the initiation of T-DXd, the treatment was classified as the T-DXd(−) group for that specific endpoint, as the drug could not have influenced the outcome. Consequently, the number of treatments classified as T-DXd(+) differed between the safety analysis (n = 137) and the efficacy analysis (n = 61), reflecting cases where T-DXd was initiated after intracranial progression but prior to potential late radiation toxicities.

Additionally, to address potential selection bias concerns regarding the timing of drug administration, a subgroup analysis was performed based on the temporal proximity of T-DXd to SRS. The concurrent T-DXd group was defined as patients who received T-DXd within 3 months of the SRS procedure. Patients who received T-DXd outside this 3-month window were classified as the non-concurrent T-DXd group.

### 2.3 Definition of analysis levels

To comprehensively evaluate both systemic survival outcomes and lesion-specific responses, analyses were performed at two predefined levels. Patient-level analyses were conducted in the entire cohort of 113 patients to assess overall survival (OS), RN, radionecrosis-free survival (RNFS), and baseline demographic and clinical characteristics. Treatment-level analyses were performed on 461 individual SRS courses to evaluate RN and intracranial efficacy outcomes, including local failure and distant intracranial failure, in relation to lesion-specific dosimetric parameters and tumor characteristics.

### 2.4 Definition of endpoints

The diagnosis of RN was determined through a review by neuro-radiologists and attending physicians, based on the evaluation of serial brain MRI features and the presence of RN-related clinical symptoms. Differentiation between RN and tumor progression was aided by advanced imaging modalities (e.g., perfusion MRI) whenever available. The cumulative incidence of RN was calculated from the date of the corresponding SRS procedure to the date of definitive RN diagnosis. In contrast, for patients who did not develop RN, the observation period was censored at the last follow-up, calculated from the date of the initial SRS. Similarly, for RNFS, defined as the time to the first occurrence of either RN or death from any cause, the time interval was measured from the date of the corresponding SRS for patients who developed RN, whereas it was measured from the date of the initial SRS for those without RN.

To evaluate intracranial efficacy, intracranial progression was categorized into local failure and distant intracranial failure. Time-to-event intervals were calculated from the date of SRS. Local failure was defined as radiographic progression of the treated lesion within the SRS target volume. Distant intracranial failure was defined as the appearance of new brain metastases or the progression of non-irradiated lesions outside the SRS field. Any intracranial progression was defined as the occurrence of either local failure or distant intracranial failure.

For the specific subgroup analysis of patients in the T-DXd(+) group, clinical outcomes including any intracranial progression-free survival and OS were calculated from the date of T-DXd initiation, distinct from the primary analysis which utilized the date of SRS as the baseline.

### 2.5 Statistical analysis

All statistical analyses were performed using R Studio (version 4.3.3; R Foundation for Statistical Computing, Vienna, Austria). The cumulative incidence rates of RN and intracranial progression were estimated using the cumulative incidence function, with death considered as a competing risk. Survival outcomes were estimated using the Kaplan-Meier method, and differences were assessed using the log-rank test. For univariate and multivariate analyses, the Fine and Gray competing risks regression model was employed for outcomes subject to competing risks, while the Cox proportional hazards model was used for survival endpoints. Variables with a *p* value less than 0.2 in the univariate analysis were included in the multivariate analysis.

To minimize selection bias and account for potential confounding by indication, a propensity score matching (PSM) analysis was performed. Patients in the T-DXd group were matched 1:1 with those in the control group using the nearest neighbor method without replacement. The matching covariates included variables that showed significant imbalances in the primary analysis: tumor volume, prescription dose, fractionation schedule (single vs. fractionated), and history of prior brain surgery.

### 2.6 Ethics statement

The study protocol was reviewed and approved by the Institutional Review Board (IRB) of each participating center. The requirement for informed consent was waived by the IRBs due to the retrospective nature of the study and the use of de-identified data.

## 3 Results

A total of 113 patients who underwent SRS for HER2-positive or HER2-low BCBM were included in this retrospective analysis. Among them, 29 patients (25.7%) were classified into the T-DXd(+) group, while 84 patients (74.3%) comprised the T-DXd(−) group. The baseline characteristics of the study population are summarized in Table 1. Baseline characteristics were generally well-balanced between the two cohorts, with the exception of a history of brain surgery. The proportion of patients who had undergone prior brain surgery was significantly higher in the T-DXd(−) group compared with the T-DXd(+) group (29.8% vs. 6.9%, *p* = 0.025). Other treatment-related factors, including prior whole brain radiation therapy (WBRT) history, number of treated lesions, multiple SRS courses or repeated SRS, did not differ significantly between the groups. The mean follow-up duration was 28.7 ± 23.3 months, which was similar for both groups (*p* = 0.750).

**Table 1:** Clinical and treatment characteristics of patients treated with SRS, stratified by T-DXd status.

	Total Patients (N = 113)	T-DXd (−) (N = 84)	T-DXd (+) (N = 29)	*p* value
**Age (years)**				
Mean ± SD	55.7 ± 8.9	56.0 ± 8.5	54.8 ± 9.9	0.516
<50	24 (21.2%)	16 (19.0%)	8 (27.6%)	0.480
≥50	89 (78.8%)	68 (81.0%)	21 (72.4%)	
**Hormone Receptor Status**				
**ER**				0.495
Positive	47 (41.6%)	37 (44.0%)	10 (34.5%)	
Negative	66 (58.4%)	47 (56.0%)	19 (65.5%)	
**PR**				0.988
Positive	33 (29.2%)	24 (28.6%)	9 (31.0%)	
Negative	80 (70.8%)	60 (71.4%)	20 (69.0%)	
**HER2 Status**				0.999
Positive (High)	99 (87.6%)	74 (88.1%)	25 (86.2%)	
Low	14 (12.4%)	10 (11.9%)	4 (13.8%)	
Treatment History				
**History of Brain Surgery**				0.025
Yes	27 (23.9%)	25 (29.8%)	2 (6.9%)	
No	86 (76.1%)	59 (70.2%)	27 (93.1%)	
**History of WBRT**				0.999
Yes	39 (34.5%)	29 (34.5%)	10 (34.5%)	
No	74 (65.5%)	55 (65.5%)	19 (65.5%)	
**SRS Characteristics**				
**Number of Treated Lesions**				0.974
Multiple	64 (56.6%)	47 (56.0%)	17 (58.6%)	
Single	49 (43.4%)	37 (44.0%)	12 (41.4%)	
**Multiple SRS Courses**^a^				0.640
Yes	64 (56.6%)	46 (54.8%)	18 (62.1%)	
No	49 (43.4%)	38 (45.2%)	11 (37.9%)	
**Repeated SRS**^b^				0.904
Yes	36 (31.9%)	26 (31.0%)	10 (34.5%)	
No	77 (68.1%)	58 (69.0%)	19 (65.5%)	
**Use of T-DM1**				0.391
Yes	68 (60.2%)	53 (63.1%)	15 (51.7%)	
No	45 (39.8%)	31 (36.9%)	14 (48.3%)	
**Follow-up (mo)**				
Mean ± SD	28.7 ± 23.3	29.0 ± 26.0	27.8 ± 12.9	0.750

Abbreviations: ER, estrogen receptor; HER2, human epidermal growth factor receptor 2; mo, months; PR, progesterone receptor; SD, standard deviation; SRS, stereotactic radiosurgery; T-DM1, trastuzumab emtansine; T-DXd, trastuzumab deruxtecan; WBRT, whole brain radiation therapy.

a SRS delivered in separate treatment courses (distinct from fractionated SRS).

b Repeated SRS delivered to a previously treated lesion.

**Fig. 1:**
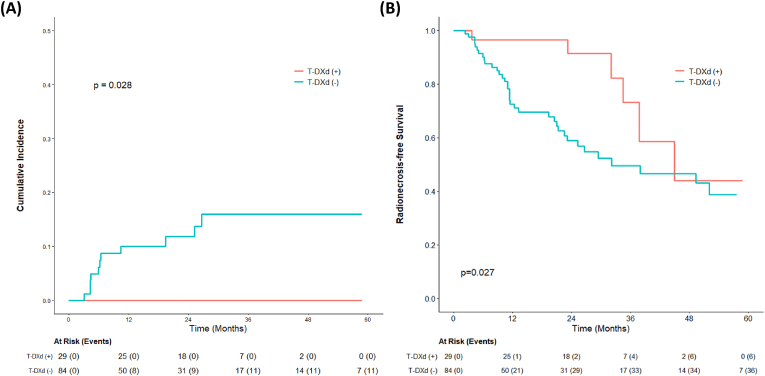
**Patient-level analysis of radionecrosis outcomes according to T-DXd status (n=113 patients).** (A) Cumulative incidence of radionecrosis. (B) Radionecrosis-free survival. *Abbreviations: T-DXd, trastuzumab deruxtecan*.

For the evaluation of RN, no cases were observed in the T-DXd(+) group during the follow-up period, whereas 11 events occurred in the T-DXd(−) group. The estimated 1-year and 2-year cumulative incidence rates of RN were 10% and 14% for the T-DXd(−) group, respectively, compared with 0% for the T-DXd(+) group at both time points. This difference in cumulative incidence was statistically significant (Fig. 1A, *p* = 0.028). Univariate and multivariate analyses were performed to identify factors associated with the risk of RN (Supplementary Table 1). In the univariate analysis, the use of T-DXd was significantly associated with a reduced risk of RN events (*p* < 0.001); however, the HR was not estimable due to the complete absence of events in the T-DXd(+) arm. Other factors, including age, subtype, and SRS characteristics, did not show statistically significant associations, although estrogen receptor (ER) positivity showed a trend toward increased risk (*p* = 0.068). We further evaluated the impact of specific anti-HER2 agents across the T-DXd(−) cohort to characterize the background therapies of patients who developed RN. No other single agent demonstrated a statistically significant association with RN risk in this cohort (Supplementary Table 2).

Regarding RNFS, the T-DXd(+) group demonstrated superior outcomes compared with the T-DXd(−) group (Fig. 1B, *p* = 0.027). The estimated 1-year and 2-year RNFS rates were 89.3% and 67.9% for the T-DXd(+) group, respectively, compared with 73.7% and 53.3% for the T-DXd(−) group. In the multivariate Cox proportional hazards analysis (Table 2), use of T-DXd remained significantly associated with improved RNFS (HR 0.31; 95% CI, 0.13–0.75; *p* = 0.009). Additionally, a history of brain surgery was associated with improved RNFS (HR 0.43; 95% CI, 0.20–0.95; *p* = 0.036).

**Table 2:** Univariate and multivariate analysis of factors associated with radionecrosis-free survival (patient-level analysis).

Factors		Univariate		Multivariate	
		HR (95% CI)	*p* value	HR (95% CI)	*p* value
**Age**	(<50 vs ≥ 50)	1.23 (0.57-2.65)	0.599		
**ER**	(No vs Yes)	0.99 (0.54-1.81)	0.964		
**PR**	(No vs Yes)	0.65 (0.33-1.28)	0.215		
**HER2**	(High vs Low)	1.42 (0.63-3.19)	0.402		
**History of Brain Surgery**	(No vs Yes)	0.54 (0.25-1.17)	0.119	**0.43 (0.20**–**0.95)**	**0.036**
**History of WBRT**	(No vs Yes)	1.36 (0.74-2.51)	0.317		
**Number of Treated Lesions**	(Single vs Multiple)	1.27 (0.69-2.33)	0.444		
**Multiple SRS Courses**^a^	(No vs Yes)	0.79 (0.42-1.48)	0.460		
**Repeated SRS**^b^	(No vs Yes)	0.70 (0.37-1.32)	0.274		
**Use of T-DM1**	(No vs Yes)	1.67 (0.87-3.20)	0.125	1.49 (0.77-2.86)	0.236
**Use of T-DXd**	(No vs Yes)	**0.37 (0.16**–**0.88)**	**0.025**	**0.31 (0.13**–**0.75)**	**0.009**

Abbreviations: CI, confidence interval; ER, estrogen receptor; HER2, human epidermal growth factor receptor 2; HR, hazard ratio; PR, progesterone receptor; SRS, stereotactic radiosurgery; T-DM1, trastuzumab emtansine; T-DXd, trastuzumab deruxtecan; WBRT, whole brain radiation therapy.

a SRS delivered in separate treatment courses (distinct from fractionated SRS).

b Repeated SRS delivered to a previously treated lesion.

We further evaluated the clinical outcomes of the 29 patients in the T-DXd(+) group, with survival calculated from the date of T-DXd initiation to provide a perspective on the systemic therapy's efficacy. The Kaplan-Meier estimates for any intracranial progression-free survival and OS are presented in Supplementary Fig. 1. The estimated 1-year and 2-year any intracranial progression-free survival rates were 60.9% and 22.9%, respectively. Furthermore, the 1-year and 2-year OS rates were 86.8% and 62.0%, respectively.

**Fig. 2:**
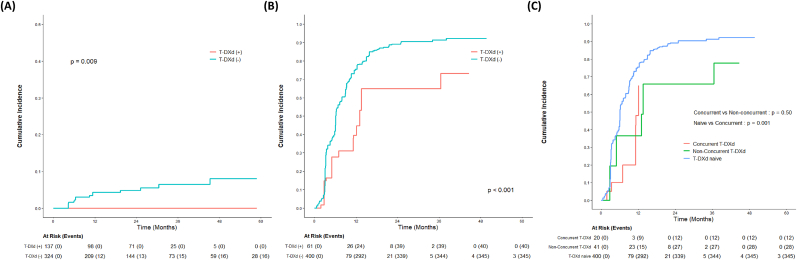
**Treatment-level analysis of radionecrosis and intracranial efficacy outcomes according to T-DXd status (n=461 treatments).** (A) Cumulative incidence of radionecrosis. (B) Cumulative incidence of any intracranial progression. (C) Cumulative incidence of any intracranial progression stratified by the timing of T-DXd administration. *Abbreviations: T-DXd, trastuzumab deruxtecan*.

In the treatment-level analysis of 461 SRS courses, consisting of 61 treatments (13.2%) in the T-DXd(+) group and 400 treatments (86.8%) in the T-DXd(−) group, patient-related factors remained well-balanced (Table 3). The T-DXd(+) group was characterized by a significantly lower history of brain surgery (1.6% vs. 23.0%, *p* < 0.001), smaller treatment volumes (*p* = 0.001), and more frequent use of single-fraction SRS (85.2% vs. 64.0%, *p* = 0.002) with lower radiation dose (p = 0.001). The mean follow-up duration was significantly shorter in the T-DXd(+) group compared with the T-DXd(−) group (15.5 vs. 28.1 months, *p* < 0.001). Consistent with the patient-level analysis, the treatment-level analysis demonstrated a significantly lower risk of RN in the T-DXd(+) group (Fig. 2A, *p* = 0.009). The 1-year and 2-year cumulative incidence rates of RN were 0% in the T-DXd(+) group, compared with 4.3% and 4.9% in the T-DXd(−) group, respectively.

**Table 3:** Clinical and treatment characteristics of SRS courses, stratified by T-DXd status.

	Total Treatments (N = 461)	T-DXd (−) (N = 400)	T-DXd (+) (N = 61)	*p* value
**Age (years)**				
Mean ± SD	57.0 ± 8.6	57.1 ± 8.6	56.2 ± 8.4	0.442
<50	75 (16.3%)	67 (16.8%)	8 (13.1%)	0.596
≥50	386 (83.7%)	333 (83.2%)	53 (86.9%)	
**Hormone Receptor Status**				
**ER**				0.414
Positive	169 (36.7%)	150 (37.5%)	19 (31.1%)	
Negative	292 (63.3%)	250 (62.5%)	42 (68.9%)	
**PR**				0.489
Positive	123 (26.7%)	104 (26.0%)	19 (31.1%)	
Negative	338 (73.3%)	296 (74.0%)	42 (68.9%)	
**HER2 Status**				0.324
Positive (High)	410 (88.9%)	353 (88.2%)	57 (93.4%)	
Low	51 (11.1%)	47 (11.8%)	4 (6.6%)	
**Treatment History**				
**History of Brain Surgery**				<0.001
Yes	93 (20.2%)	92 (23.0%)	1 (1.6%)	
No	368 (79.8%)	308 (77.0%)	60 (98.4%)	
**History of WBRT**				0.509
Yes	235 (51.0%)	201 (50.2%)	34 (55.7%)	
No	226 (49.0%)	199 (49.8%)	27 (44.3%)	
**SRS Characteristics**				
**SRS Fractionation**				0.002
Multiple	153 (33.2%)	144 (36.0%)	9 (14.8%)	
Single	308 (66.8%)	256 (64.0%)	52 (85.2%)	
**SRS Dose (Gy)**				0.001
Mean ± SD	22.9 ± 5.6	23.2 ± 5.7	21.2 ± 4.0	
**Treatment Volume (cm^3^)**				<0.001
Mean ± SD	1.9 ± 4.6	2.1 ± 4.9	0.5 ± 0.9	
**Number of Treated Lesions**				0.942
Multiple	403 (87.4%)	349 (87.2%)	54 (88.5%)	
Single	58 (12.6%)	51 (12.8%)	7 (11.5%)	
**Repeated SRS**^a^				0.800
Yes	77 (16.7%)	68 (17.0%)	9 (14.8%)	
No	384 (83.3%)	332 (83.0%)	52 (85.2%)	
**Follow-up (mo)**				
Mean ± SD	26.4 ± 20.7	28.1 ± 21.5	15.5 ± 8.9	<0.001

Abbreviations: ER, estrogen receptor; Gy, Gray; HER2, human epidermal growth factor receptor 2; mo, months; PR, progesterone receptor; SD, standard deviation; SRS, stereotactic radiosurgery; T-DXd, trastuzumab deruxtecan; WBRT, whole brain radiation therapy.

a Repeated SRS delivered to a previously treated lesion.

Given the baseline imbalances in prognostic factors, we performed a PSM analysis to validate the safety findings. The matching process yielded a well-balanced cohort of 134 pairs, with no significant differences in tumor volume, radiation dose, fractionation, or surgical history between the T-DXd and matched control groups (Supplementary Table 3). While the cumulative incidence of RN did not differ significantly between the groups, a trend toward a reduced risk was observed in the T-DXd group (p = 0.077) (Supplementary Fig. 2).

In terms of intracranial efficacy, the T-DXd(+) group demonstrated superior outcomes across all evaluated endpoints. The cumulative incidence of any intracranial progression was significantly lower in the T-DXd(+) group compared with the T-DXd(−) group (Fig. 2B, *p* < 0.001); the estimated 1-year cumulative incidence rates were 40% vs. 76%, respectively. Similarly, the T-DXd(+) group showed superior local control with a 1-year local failure rate of 6.6%, compared with 29% in the T-DXd(−) group (Supplementary Fig. 3A and p = 0.002). Consistent with these findings, the cumulative incidence of distant intracranial failure was also significantly reduced in the T-DXd(+) group (Supplementary Fig. 3B and p = 0.009), with 1-year rates of 40% vs. 66%.

Multivariate analysis confirmed that T-DXd use was significantly associated with a reduced risk of any intracranial progression (Table 4; HR 0.39; 95% CI, 0.27–0.56; *p* < 0.001), along with history of WBRT, single fractionation SRS, number of treated lesions, and repeated SRS. When analyzed by failure pattern, T-DXd maintained its significance as a factor associated with a reduced risk for both local failure (Supplementary Table 4; HR 0.33; 95% CI, 0.16–0.66; *p* = 0.002) and distant intracranial failure (Supplementary Table 5; HR 0.50; 95% CI, 0.35–0.72; *p* < 0.001). Conversely, a history of WBRT was identified as a significant risk factor for both any intracranial progression and distant intracranial failure.

**Table 4:** Univariate and multivariate analysis of factors associated with any intracranial progression (treatment-level analysis).

Factors		Univariate		Multivariate	
		HR (95% CI)	*p* value	HR (95% CI)	*p* value
**Age**	(<50 vs ≥ 50)	1.08 (0.82-1.41)	0.580		
**ER**	(No vs Yes)	0.96 (0.78-1.18)	0.680		
**PR**	(No vs Yes)	0.91 (0.73-1.13)	0.400		
**HER2**	(High vs Low)	1.00 (0.70-1.41)	0.980		
**History of Brain Surgery**	(No vs Yes)	1.07 (0.86-1.32)	0.560		
**History of WBRT**	(No vs Yes)	**1.37 (1.12**–**1.68)**	**0.003**	**1.41 (1.14**–**1.75)**	**0.002**
**SRS Fractionation**	(Multiple vs Single)	**1.45 (1.17**–**1.78)**	**<0.001**	**1.53 (1.11**–**2.11)**	**0.010**
**SRS Dose (Gy)**	(Continuous)	**0.98 (0.96**–**0.99)**	**0.002**	1.00 (0.98-1.03)	0.980
**Treatment Volume (cm^3^)**	(Continuous)	0.98 (0.97-1.00)	0.076	1.01 (0.99-1.02)	0.360
**Number of Treated Lesions**	(Multiple vs Single)	**0.53 (0.39**–**0.71)**	**<0.001**	**0.53 (0.38**–**0.73)**	**<0.001**
**Repeated SRS**^a^	(No vs Yes)	**1.26 (1.02**–**1.54)**	**0.029**	**1.31 (1.05**–**1.64)**	**0.015**
**Use of T-DXd**	(No vs Yes)	**0.48 (0.35**–**0.67)**	**<0.001**	**0.39 (0.27**–**0.56)**	**<0.001**

Abbreviations: CI, confidence interval; ER, estrogen receptor; Gy, Gray; HER2, human epidermal growth factor receptor 2; HR, hazard ratio; PR, progesterone receptor; SRS, stereotactic radiosurgery; T-DXd, trastuzumab deruxtecan; WBRT, whole brain radiation therapy.

a Repeated SRS delivered to a previously treated lesion.

To further mitigate potential selection bias arising from the prolonged interval between SRS and T-DXd, a subgroup analysis was performed stratified by the timing of T-DXd administration. Patients receiving concurrent T-DXd (within 3 months of SRS) demonstrated a significantly lower cumulative incidence of any intracranial progression compared with T-DXd naïve patients (Fig. 2C, *p* = 0.001). Furthermore, the efficacy of concurrent T-DXd was comparable to that of non-concurrent T-DXd (*p* = 0.50).

## 4 Discussion

In this multi-center retrospective study, we report two principal findings regarding the combination of T-DXd and SRS for patients with HER2-positive or HER2-low BCBM. First, contrary to the theoretical concerns derived from other ADCs, T-DXd was not associated with an increased risk of RN. In fact, the T-DXd group demonstrated a favorable safety profile with no observed cases of RN in both patient-level and treatment-level analyses during the study period. Second, T-DXd was associated with superior intracranial efficacy after multivariable adjustment. The use of T-DXd was associated with a reduced risk of any intracranial progression, local failure, and distant intracranial failure, with these benefits remaining robust in multivariate analyses. Collectively, these findings suggest that T-DXd can be safely combined with SRS, potentially contributing to improved intracranial control without compromising neurological safety.

While the intracranial efficacy of ADCs is well-recognized, the potential for synergistic toxicity with SRS—particularly RN—remains a critical concern [6,7]. Lebow et al. reported an association between concurrent ADC administration and increased symptomatic necrosis; however, this finding must be interpreted with caution [6]. In their study, T-DM1 and T-DXd each constituted approximately half of the ADC cohort, yet no subgroup analysis was conducted to differentiate the risk between agents. Similarly, a recent multi-institutional Japanese study reported increased symptomatic RN with concurrent ADC use [7]. Notably, within their concurrent ADC subset (n = 19), the vast majority of patients received T-DM1 (n = 15), while only a minority received T-DXd (n = 4). Given the robust evidence linking T-DM1 to radiation injury [[9], [10], [11], [12]], it is possible that the high proportion of T-DM1 use in these studies may partly account for the reported increase in RN risk. In our cohort, prior T-DM1 exposure was not statistically associated with increased RN risk. This exploratory finding should be interpreted cautiously, as the limited number of RN events and variability in administration timing relative to SRS may have reduced the ability to detect agent-specific effects.

Our observations align with the recent multi-institutional study by Khatri et al., which reported favorable outcomes for concurrent T-DXd and SRS [14]. Their study demonstrated a safety profile comparable to historical SRS data, with 2-year cumulative RN rates of 11% per patient and 2.1% per lesion. Additionally, they reported excellent local control rates of 97% and 88% at 1 and 2 years, respectively. Similarly, data from a European multi-center study demonstrated a favorable safety profile for T-DXd combined with SRS, reporting symptomatic RN in just 2.7% of patients (1/37) and no significant impact on T-DXd discontinuation rates compared to the T-DXd alone group [15]. While corroborating these favorable safety and efficacy profiles, our study provides a distinct contribution by directly comparing T-DXd with a control group receiving other anti-HER2 therapies.

Beyond corroborating a favorable safety profile regarding RN, our study suggests a potential therapeutic synergy between SRS and T-DXd, supported by the radiobiological mechanism of the payload. Mechanistically, this synergy is likely driven by the payload of T-DXd, deruxtecan (DXd), a potent topoisomerase I inhibitor. Unlike the payload of T-DM1, which targets microtubules, topoisomerase I inhibitors are well-established radiosensitizers [16,17]. They stabilize the DNA-topoisomerase I cleavable complex and inhibit the repair of radiation-induced DNA single-strand breaks, thereby converting them into lethal double-strand breaks. Additionally, the high membrane permeability of the DXd payload exerts a bystander effect, potentially targeting antigen-low or hypoxic tumor cells surrounding the irradiated volume [18,19]. While direct prospective evidence for this specific combination remains evolving, data from several recent studies support this potential benefit. The DEBBRAH trial and ROSET-BM study have highlighted the durable intracranial activity of T-DXd, even in patients with prior SRS. Furthermore, the TENDENCE study reported minimal toxicity (Grade 1 RN only) in patients treated with concurrent T-DXd and radiotherapy [[20], [21], [22], [23]].

Collectively, these findings, along with the well-defined superior intracranial activity of T-DXd [[24], [25], [26], [27], [28]], suggest that its combination with SRS likely offers a dual mechanism of action: SRS provides immediate local ablation of macroscopic disease, while T-DXd maintains systemic and intracranial control, potentially suppressing the seeding of new metastases. This potential synergistic effect aligns with and may explain the superior local and distant intracranial control rates observed in our current study.

However, interpretation of our safety results requires caution regarding potential confounders. Notably, the T-DXd group was characterized by significantly smaller treatment volumes and lower radiation doses compared to the control group. We attribute this largely to selection bias and differences in surveillance intensity; patients receiving T-DXd in contemporary practice likely undergo more rigorous MRI monitoring, facilitating the detection of brain metastases at an earlier, asymptomatic stage. Consequently, the absence of RN events in the T-DXd cohort may be partially driven by these favorable dosimetric factors rather than the pharmacological properties of T-DXd alone. Nevertheless, to rigorously address this issue, we conducted a sensitivity analysis using PSM. Even after balancing for treatment volume, dose, and fractionation, the T-DXd group did not demonstrate an increased risk of RN compared to the matched control group. This consistency across both the unadjusted and matched analyses strengthens the conclusion that T-DXd can be safely combined with SRS.

Our study is subject to limitations inherent to its retrospective design. First, the relatively small sample size and limited number of RN events may have reduced statistical power, particularly for subgroup or agent-specific analyses. Second, the follow-up duration for the T-DXd group was significantly shorter than that of the control group (15.5 vs. 28.1 months). Since RN is a late-onset toxicity with a latency period that can exceed one year, the absence of RN in the T-DXd arm must be interpreted with caution, as the observation window may have been insufficient to capture late events. Third, the lack of central radiologic review in this retrospective analysis may have introduced inter-institutional variability in the diagnosis of RN. Consequently, diagnostic accuracy relied on local clinical practice without standardized implementation of advanced imaging or histologic confirmation, potentially limiting the uniformity of diagnostic thresholds. While our findings indicate a favorable safety profile, long-term risk assessment warrants validation through larger prospective studies with extended monitoring.

In conclusion, our findings suggest that the combination of T-DXd and SRS is not associated with a significant increase in the risk of radionecrosis in patients with HER2-positive or HER2-low breast cancer brain metastases. Moreover, the superior intracranial control observed—manifested by reduced rates of both local and distant intracranial failures—indicates that the combination of SRS with T-DXd represents a potentially effective therapeutic strategy with a favorable safety profile. Further prospective investigations are warranted to validate these results and optimize treatment sequencing.

## CRediT authorship contribution statement

**Seok-Joo Chun:** Writing – original draft, Visualization, Software, Resources, Methodology, Formal analysis, Data curation. **Kyubo Kim:** Writing – review & editing, Writing – original draft, Validation, Supervision, Project administration, Data curation, Conceptualization. **Won Ick Chang:** Writing – review & editing, Investigation, Data curation. **Yong Bae Kim:** Writing – review & editing, Investigation, Data curation. **Sun Ha Paek:** Writing – review & editing, Investigation, Data curation. **Kyung-Hun Lee:** Writing – review & editing, Investigation, Data curation. **Jin-Ho Song:** Writing – review & editing, Investigation, Data curation. **Ji Hyun Hong:** Writing – review & editing, Investigation, Data curation. **Jieun Lee:** Writing – review & editing, Investigation, Data curation. **Won Il Jang:** Writing – review & editing, Investigation, Data curation. **Tae Hyun Kim:** Writing – review & editing, Investigation, Data curation. **Kyung Hwan Shin:** Writing – review & editing, Validation, Supervision, Resources, Project administration, Funding acquisition, Conceptualization.

## Ethics approval

The study protocol was reviewed and approved by the Institutional Review Board (IRB) of each participating center.

## Funding

This work was supported by the National R&D Program for Cancer Control through the 10.13039/501100003645National Cancer Center (NCC) funded by the 10.13039/501100003625Ministry of Health & Welfare, Republic of Korea (HA22C0044).

## Funding resources

None.

## Declaration of competing interest

The authors declare the following financial interests/personal relationships which may be considered as potential competing interests: Kyung Hwan Shin reports administrative support and article publishing charges were provided by Korea Ministry of Health and Welfare. The other authors declare that they have no known competing financial interests or personal relationships that could have appeared to influence the work reported in this paper
