# Supplementary material for: Impact of trastuzumab deruxtecan (T-DXd) and brain stereotactic radiosurgery on intracranial control and radionecrosis risk in HER2-positive or -low breast cancer brain metastases
Source: Breast. 2026 Mar 17;87:104751. doi: 10.1016/j.breast.2026.104751 (PMC13049524; doi:10.1016/j.breast.2026.104751)
Supplement: Multimedia component 1 [file mmc1.docx]

****Supplementary Table 1. Univariate and Multivariate Analysis of Factors Associated with Radionecrosis (Patient-level Analysis)****

| **Factors** | | Univariate | | Multivariate | |
| --- | --- | --- | --- | --- | --- |
|  |  | HR (95% CI) | *p* value | HR (95% CI) | *p* value |
| **Age** | (<50 vs >=50) | 1.36 (0.29-6.38) | 0.700 |  |  |
| **ER** | (No vs Yes) | **2.98 (0.92-9.66)** | **0.068** | 2.80 (0.88-8.94) | 0.082 |
| **PR** | (No vs Yes) | 1.03 (0.31-3.43) | 0.960 |  |  |
| **HER2** | (High vs Low) | 1.45 (0.31-6.77) | 0.640 |  |  |
| **History of Brain Surgery** | (No vs Yes) | 0.55 (0.13-2.30) | 0.410 |  |  |
| **History of WBRT** | (No vs Yes) | 0.92 (0.28-3.07) | 0.890 |  |  |
| **Number of Treated Lesions** | (Single vs Multiple) | 1.21 (0.38-3.85) | 0.750 |  |  |
| **Multiple SRS Courses^†^** | (No vs Yes) | 1.72 (0.52-5.68) | 0.370 |  |  |
| **Repeated SRS**^§^ | (No vs Yes) | 1.27 (0.43-3.70) | 0.670 |  |  |
| **Use of T-DM1** | (No vs Yes) | 0.93 (0.30-2.86) | 0.890 |  |  |
| **Use of T-DXd** | (No vs Yes) | **n/a*** | <0.001 | **n/a*** | <0.001 |

*Abbreviations: CI, confidence interval; ER, estrogen receptor; HER2, human epidermal growth factor receptor 2; HR, hazard ratio; n/a, not applicable; PR, progesterone receptor; SRS, stereotactic radiosurgery; T-DM1, trastuzumab emtansine; T-DXd, trastuzumab deruxtecan; WBRT, whole brain radiation therapy.***† SRS delivered in separate treatment courses (distinct from fractionated SRS).
§ Repeated SRS delivered to a previously treated lesion*** Hazard ratios for T-DXd were not estimable (n/a) due to the absence of radionecrosis events in the T-DXd(+) group.

# **Supplementary Table 2. Association Between Prior Anti-HER2 Therapy Exposure and Radionecrosis in the T-DXd(–) Group**

| **Anti-HER2 agents** | **Exposed** | | | **Unexposed** | | | **OR^‡^** | ***p* value** |
| --- | --- | --- | --- | --- | --- | --- | --- | --- |
|  | **Total** | **RN (+)** | **Incidence** | **Total** | **RN (+)** | **Incidence** |  |  |
| Trastuzumab | 83 | 11 | 13.3% | 1 | 0 | 0.0% | n/a | n/a |
| Pertuzumab | 53 | 8 | 15.1% | 31 | 3 | 9.7% | 1.66  (0.41-6.78) | 0.481 |
| Lapatinib | 34 | 4 | 11.8% | 50 | 7 | 14.0% | 0.82  (0.22-3.05) | 0.766 |
| T-DM1 | 53 | 6 | 11.3% | 31 | 5 | 16.1% | 0.66  (0.18-2.39) | 0.530 |
| Other anti-HER2 agents**^†^** | 3 | 0 | 0.0% | 81 | 11 | 13.6% | n/a | n/a |

**Abbreviations: CI, confidence interval; n/a, not applicable; OR, odds ratio; RN, radionecrosis.**

**† Other anti-HER2 agents consisted of Phesgo (n=2) and tucatinib (n=1).**

**‡ Calculated using univariate logistic regression (exposed vs. unexposed).**

Note: Categories are not mutually exclusive as patients may have received multiple lines of anti-HER2 therapy. OR for Trastuzumab was not estimable due to the lack of events in the unexposed group.

# **Supplementary Table 3. Clinical and Treatment Characteristics of SRS Courses in the Propensity Score-Matched Cohort**

|  |  | **T-DXd (-)** | **T-DXd (+)** | *p* value |
| --- | --- | --- | --- | --- |
|  |  | **(N=134)** | **(N=134)** |  |
| **Age (years)** | |  |  | 0.005 |
| Mean ± SD | | 57.9 ± 8.6 | 54.8 ± 9.4 |  |
| **Hormone Receptor Status** | |  |  |  |
| **ER** | |  |  | 0.421 |
| Positive | | 36 (26.9%) | 43 (32.1%) |  |
| Negative | | 98 (73.1%) | 91 (67.9%) |  |
| **PR** | |  |  | 0.197 |
| Positive | | 27 (20.1%) | 37 (27.6%) |  |
| Negative | | 107 (79.9%) | 97 (72.4%) |  |
| **HER2 Status** | |  |  | 0.191 |
| Positive (High) | | 126 (94.0%) | 119 (88.8%) |  |
| Low | | 8 (6.0%) | 15 (11.2%) |  |
| **Treatment History** | |  |  |  |
| **History of Brain Surgery** | |  |  | 0.999 |
| Yes | | 7 (5.2%) | 7 (5.2%) |  |
| No | | 127 (94.8%) | 127 (94.8%) |  |
| **History of WBRT** | |  |  | 0.903 |
| Yes | | 67 (50.0%) | 65 (48.5%) |  |
| No | | 67 (50.0%) | 69 (51.5%) |  |
| **SRS Characteristics** | |  |  |  |
| **SRS Fractionation** | |  |  | 0.426 |
| Multiple | | 21 (15.7%) | 27 (20.1%) |  |
| Single | | 113 (84.3%) | 107 (79.9%) |  |
| **SRS Dose (Gy)** | |  |  | 0.587 |
| Mean ± SD | | 21.5 ± 4.7 | 21.8 ± 4.7 |  |
| **Treatment Volume (cm^3^)** | |  |  | 0.719 |
| Mean ± SD | | 0.9 ± 1.8 | 0.8 ± 1.6 |  |
| **Number of Treated Lesions** | |  |  | 0.836 |
| Multiple | | 122 (91.0%) | 120 (89.6%) |  |
| Single | | 12 (9.0%) | 14 (10.4%) |  |
| **Repeated SRS^†^** | |  |  | 0.614 |
| Yes | | 19 (14.2%) | 23 (17.2%) |  |
| No | | 115 (85.8%) | 111 (82.8%) |  |
| **Follow-up (mo)** | |  |  | 0.009 |
| Mean ± SD | | 29.8 ± 22.8 | 23.8 ± 13.1 |  |

*Abbreviations: ER, estrogen receptor; Gy, Gray; HER2, human epidermal growth factor receptor 2; mo, months; PR, progesterone receptor; SD, standard deviation; SRS, stereotactic radiosurgery; T-DXd, trastuzumab deruxtecan; WBRT, whole brain radiation therapy.*

**Notes:
* Propensity score matching was performed using the following variables: history of brain surgery, SRS fractionation, SRS dose, GTV volume, repeated SRS, and history of WBRT.
* Patients who progressed before T-DXd administration were classified into the T-DXd (-) group for the efficacy analysis (time-dependent covariate adjustment)
†Repeated SRS delivered to a previously treated lesion.**

| **Factors** | | Univariate | |  | Multivariate | |
| --- | --- | --- | --- | --- | --- | --- |
|  |  | HR (95% CI) | *p* value |  | HR (95% CI) | *p* value |
| **Age** | (<50 vs ≥50) | **3.64 (1.82-7.25)** | **<0.001** |  | **2.76 (1.39-5.46)** | **0.004** |
| **ER** | (No vs Yes) | **0.59 (0.42-0.84)** | **0.003** |  | 0.92 (0.60-1.41) | 0.720 |
| **PR** | (No vs Yes) | **0.42 (0.28-0.63)** | **<0.001** |  | **0.53 (0.32-0.87)** | **0.012** |
| **HER2** | (High vs Low) | **0.26 (0.10-0.62)** | **0.003** |  | 0.43 (0.17-1.09) | 0.074 |
| **History of Brain Surgery** | (No vs Yes) | 1.06 (0.73-1.53) | 0.760 |  |  |  |
| **History of WBRT** | (No vs Yes) | 0.87 (0.63-1.21) | 0.410 |  |  |  |
| **SRS Fractionation** | (Multiple vs Single) | 0.91 (0.65-1.27) | 0.590 |  |  |  |
| **SRS Dose (Gy)** | (Continuous) | 1.01 (0.99-1.03) | 0.330 |  |  |  |
| **Treatment Volume (cm^3^)** | (Continuous) | **1.03 (1.01-1.04)** | **0.001** |  | **1.03 (1.01-1.05)** | **0.002** |
| **Number of Treated Lesions** | (Multiple vs Single) | 1.17 (0.78-1.75) | 0.440 |  |  |  |
| **Repeated SRS^†^** | (No vs Yes) | n/a |  |  |  |  |
| **Use of T-DXd** | (No vs Yes) | **0.32 (0.16-0.64)** | **0.002** |  | **0.33 (0.16-0.66)** | **0.002** |

**Supplementary Table 4. Univariate and Multivariate Analysis of Factors Associated with Local failure** (Treatment-level Analysis)

***Abbreviations: CI, confidence interval; ER, estrogen receptor; Gy, Gray; HER2, human epidermal growth factor receptor 2; HR, hazard ratio; n/a, not applicable; PR, progesterone receptor; SRS, stereotactic radiosurgery; T-DXd, trastuzumab deruxtecan; WBRT, whole brain radiation therapy*** *† Repeated SRS was excluded from the analysis as it is performed for local progression by definition.*

| **Factors** | | Univariate | |  | Multivariate | |
| --- | --- | --- | --- | --- | --- | --- |
|  |  | HR (95% CI) | *p* value |  | HR (95% CI) | *p* value |
| **Age** | (<50 vs ≥50) | 0.86 (0.66-1.13) | 0.290 |  |  |  |
| **ER** | (No vs Yes) | 0.96 (0.77-1.20) | 0.730 |  |  |  |
| **PR** | (No vs Yes) | 0.98 (0.78-1.24) | 0.870 |  |  |  |
| **HER2** | (High vs Low) | 1.21 (0.86-1.69) | 0.270 |  |  |  |
| **History of Brain Surgery** | (No vs Yes) | 0.99 (0.78-1.26) | 0.960 |  |  |  |
| **History of WBRT** | (No vs Yes) | **1.75 (1.41-2.18)** | **<0.001** |  | **1.67 (1.33-2.10)** | **<0.001** |
| **SRS Fractionation** | (Multiple vs Single) | **1.51 (1.20-1.90)** | **<0.001** |  | 1.34 (0.95-1.89) | 0.094 |
| **SRS Dose (Gy)** | (Continuous) | **0.97 (0.95-0.99)** | **<0.001** |  | 1.00 (0.98-1.03) | 0.910 |
| **Treatment Volume (cm^3^)** | (Continuous) | **0.95 (0.91-0.99)** | **0.011** |  | 0.99 (0.95-1.02) | 0.480 |
| **Number of Treated Lesions** | (Multiple vs Single) | **0.42 (0.29-0.60)** | **<0.001** |  | **0.49 (0.32-0.73)** | **<0.001** |
| **Repeated SRS^†^** | (No vs Yes) | 0.87 (0.66-1.15) | 0.330 |  |  |  |
| **Use of T-DXd** | (No vs Yes) | **0.66 (0.48-0.90)** | **0.009** |  | **0.50 (0.35-0.72)** | **<0.001** |

**Supplementary Table 5. Univariate and Multivariate Analysis of Factors Associated with Distant Intracranial Failure** (Treatment-level Analysis)

***Abbreviations: CI, confidence interval; ER, estrogen receptor; Gy, Gray; HER2, human epidermal growth factor receptor 2; HR, hazard ratio; PR, progesterone receptor; SRS, stereotactic radiosurgery; T-DXd, trastuzumab deruxtecan; WBRT, whole brain radiation therapy.*† Repeated SRS delivered to a previously treated lesion**

**Supplementary Figure 1. Treatment outcomes in the T-DXd(+) group (n=29 patients).** (A) Any intracranial progression-free survival. (B) Overall survival.


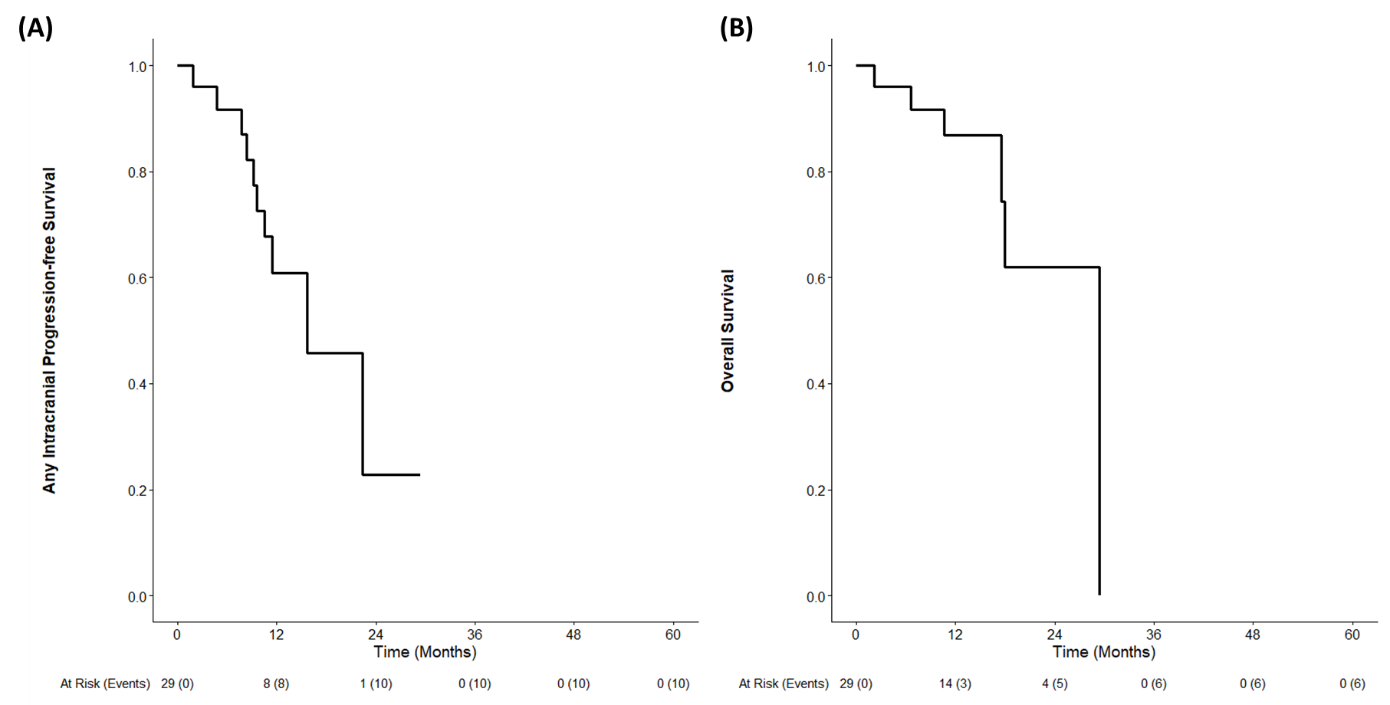


*Abbreviations: T-DXd, trastuzumab deruxtecan.*

# **Supplementary Figure 2. Treatment-level analysis of radionecrosis according to T-DXd status in the propensity score-matched cohort (n=268 treatments). Cumulative incidence of radionecrosis.**


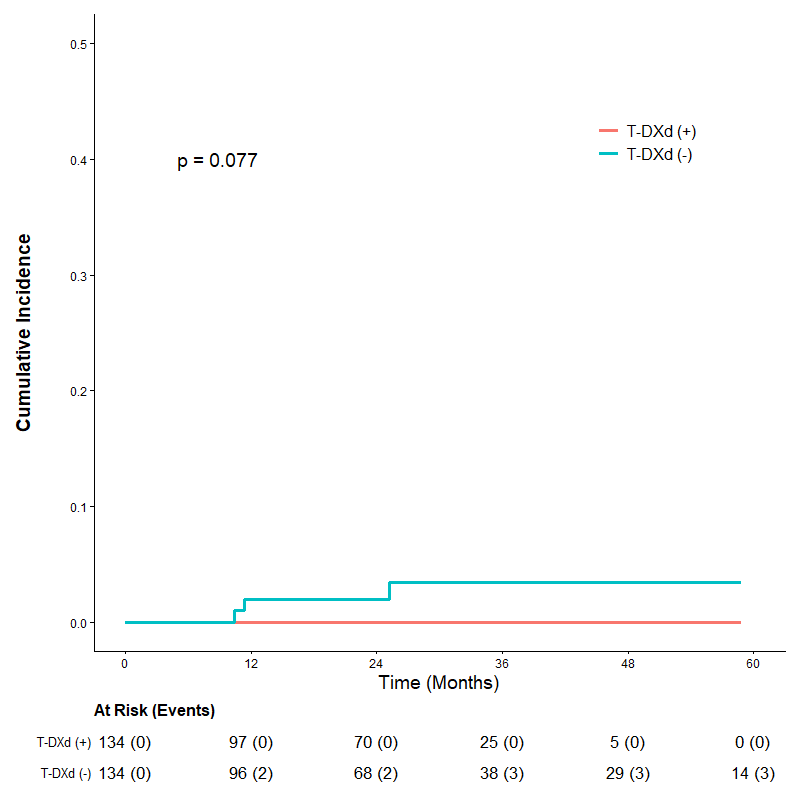


*Abbreviations: T-DXd, trastuzumab deruxtecan*

# **Supplementary Figure 3. Treatment-level analysis of detailed intracranial efficacy outcomes according to T-DXd status (n=461 treatments).** (A) Cumulative incidence of local failure. (B) Cumulative incidence of distant intracranial failure.


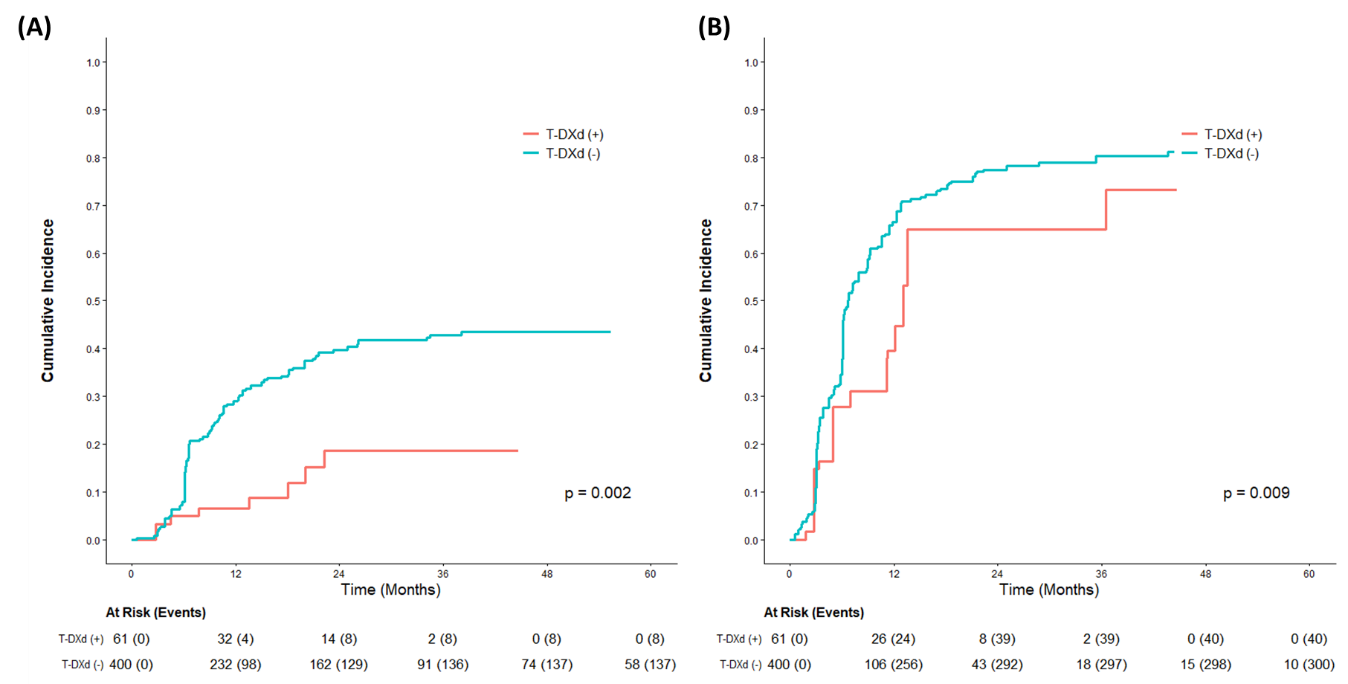


*Abbreviations: T-DXd, trastuzumab deruxtecan*
